# Ethics in scientific publications: the double copyright problem

**DOI:** 10.1590/S1808-86942010000500001

**Published:** 2015-10-22

**Authors:** Ivan D. Miziara

**Affiliations:** Associate Professor – University of São Paulo Medical School

Recently the layman's media has seen unsettling news concerning fraud in scientific publications, which included: improper conduct and data manipulation by renowned scientists.

Kremer^1^ , states that ethics in scientific publications is directly associated to the intellectual property issue - copyright, from where we have other important issues concerning the co-authorship of papers and, especially plagiarism.

However, in the daily routine of scientific journal editors there are problems which are much more mundane. Sometimes due to author's slyness. Nonetheless, most of the times I prefer to believe it is because of the lack of knowledge about the journal's rules, or even lack of attention from the authors of a scientific paper.

Kosovski^2^ states that “[…] all ethical behavior is characterized for being unpretentious and never, by mala fide or deceitful, taking credit for the deed of others”. In principle, I do not believe in bad faith, but as the saying goes: “the road to hell is paved with good intentions”, and then, authors end up making huge ethical mistakes.

Thus, just as scientific journal editors must follow COPE (Committee On Publication Ethics) rules, which developed the “Best Practice Guidelines for Journal Editors” – some sort of o Code of Ethics of those responsible for the publication of papers in a scientific journal -, authors must also be subjected to the journal's rules and, moreover, guide their conduct according to Ethics and Moral principles.

There is one aspect of this issue which is critical for its full understanding. In Brazil, as it happens elsewhere in the world, the publishing pressure has plagued the lives of researchers. Those who do not regularly publish in high-impact, or at least indexed, journals end up being left behind in the academic ranking, regardless of their intellectual capacity or their preference for teaching and not for research. This is the “publish or perish” saying, which has impacted the Brazilian Academy for some time now.

Therefore, it is to be expected that in the rush to publish and push one's scientific career forward, some researchers my make ethical mistakes. If it happens in the US and in Europe, why wouldn't it happen here?

One of the aforementioned mundane problems with which the editor has to deal is sending the same paper to two different journals at the same time.

This sometimes happens, but I wish to believe it does more because of naiveness than slyness. Nonetheless, problems arise when the author sends the paper to two different journals at the same time.

The major problem here is specifically associated with copyrights, or intellectual property. Initially, the property of a paper belongs to those who developed the research and then wrote the paper. When the paper is submitted for publication, it is implicit (and then explicit and signed) a sort of pre-contract, which states that if accepted, the author is transferring the copyright to the journal.

In exchange for the all the work the journal, its editors and revisers have of assessing the paper, it gains the right of publishing it, should it be merited.

Therefore, to send a paper to two different journals would imply in double transfer of the same copyright. This would characterize a type of intellectual fraud. It is unnecessary and redundant to comment on the ethical and moral aspects of this type of conduct.

The Brazilian Journal of Otorhinolaryngology repudiates such act and recommends that authors should wait for the final report from our revisers before submitting their papers to another journal. This is the proper and ethical conduct in this case.

## Footnotes

1 KREMER. J. M. Éticaempesquisa: um temaparareflexão. Revista da Escola de Biblioteconomia da UFMG, Belo Horizonte, v. 11, n. 12, p. 158-179, 1982.

2 KOSOVSKI, E. (Org.). Éticanacomunicação. Rio de Janeiro: Mauad, 1995.

